# Nucleation and Dynamics of Golgi-derived Microtubules

**DOI:** 10.3389/fnins.2015.00431

**Published:** 2015-11-10

**Authors:** Anna A. W. M. Sanders, Irina Kaverina

**Affiliations:** Department of Cell and Developmental Biology, Vanderbilt University Medical CenterNashville, TN, USA

**Keywords:** microtubule dynamics, Golgi-derived microtubules, γ-TuRC, CLASP, microtubule nucleation, Golgi apparatus

## Abstract

Integrity of the Golgi apparatus requires the microtubule (MT) network. A subset of MTs originates at the Golgi itself, which in this case functions as a MT-organizing center (MTOC). Golgi-derived MTs serve important roles in post-Golgi trafficking, maintenance of Golgi integrity, cell polarity and motility, as well as cell type-specific functions, including neurite outgrowth/branching. Here, we discuss possible models describing the formation and dynamics of Golgi-derived MTs. How Golgi-derived MTs are formed is not fully understood. A widely discussed model implicates that the critical step of the process is recruitment of molecular factors, which drive MT nucleation (γ-tubulin ring complex, or γ-TuRC), to the Golgi membrane via specific scaffolding interactions. Based on recent findings, we propose to introduce an additional level of regulation, whereby MT-binding proteins and/or local tubulin dimer concentration at the Golgi helps to overcome kinetic barriers at the initial nucleation step. According to our model, emerging MTs are subsequently stabilized by Golgi-associated MT-stabilizing proteins. We discuss molecular factors potentially involved in all three steps of MT formation. To preserve proper cell functioning, a balance must be maintained between MT subsets at the centrosome and the Golgi. Recent work has shown that certain centrosomal factors are important in maintaining this balance, suggesting a close connection between regulation of centrosomal and Golgi-derived MTs. Finally, we will discuss potential functions of Golgi-derived MTs based on their nucleation site location within a Golgi stack.

## Introduction

The intracellular microtubule (MT) network consisting of polarized alpha/beta tubulin polymer tubes plays important roles in intracellular trafficking, membrane dynamics, and organelle positioning. The textbook view of interphase microtubule organization is a radial array extending from a single juxtanuclear centrosome. Such organization is clearly dominant in proliferating cells where centrosome-based MTOCs are used to build mitotic spindles. Interphase cells, however, often develop non-centrosomal MT arrays. It is especially characteristic for differentiated cells that have specific function and morphology. In most specialized cell types, radial MT geometry is not ideal for precise delivery of cargos to specific cellular locations. Differentiation-related non-centrosomal microtubule networks have been described for more than 25 years; yet they remain understudied. In the last few years, the research field has started to understand the mechanisms that rearrange MTs in specialized cells and their functional significance. Non-centrosomal MT populations arise when MT-nucleating and/or stabilizing factors are concentrated at cellular scaffolds alternative to the traditional scaffold (pericentrosomal material). In many cell types the role of scaffold can be acquired by the Golgi apparatus membrane, and non-centrosomal MTs are derived from the Golgi (Chabin-Brion et al., 2001; Efimov et al., 2007; Ori-Mckenney et al., 2012; Oddoux et al., 2013). Evolutionally, it can be explained by the convenience of direct association of MT tracks with the major cellular sorting and trafficking facility. Indeed, Golgi-derived MTs were shown to support both Golgi integrity and directionality of post-Golgi trafficking (Miller et al., 2009; Hurtado et al., 2011; Vinogradova et al., 2012).

To date, Golgi-derived MTs have been characterized in hepatocytes (Chabin-Brion et al., 2001), epithelial cells (Efimov et al., 2007; Rivero et al., 2009), neurons with their strikingly diverse axonal and dendritic MT bundles (Ori-Mckenney et al., 2012; Yalgin et al., 2015), skeletal muscle (Zaal et al., 2011; Oddoux et al., 2013), and pancreatic beta cells where this MT subpopulation fine-tunes insulin secretion (Zhu et al., 2015); it is very likely that abundance of this MT sub-population will expand as we learn more about cellular architecture in differentiated tissues. Thus, the functional significance of Golgi-derived MTs is undoubted. At the same time, much remains to be elucidated about the mechanisms underlying Golgi-derived MT nucleation, stabilization, dynamics, and regulation. This review aims to give an updated view on Golgi-derived MTs, including several possible mechanisms through which Golgi-derived MTs could be formed and regulated, and how they are important for proper cell function and behavior.

## Recruitment of γ-TuRC to the Golgi membranes

The first requirement for MT formation in a cell, where tubulin concentrations are relatively low, is availability of MT nucleation templates (Oakley et al., 2015; Petry and Vale, 2015). Solid evidence indicates that MT nucleation at the Golgi starts off γ-tubulin ring complexes (γ-TuRC; (Chabin-Brion et al., 2001; Efimov et al., 2007; Ori-Mckenney et al., 2012), similar to classic centrosomal MTOC. γ-Tubulin was found associated with isolated Golgi membranes (Chabin-Brion et al., 2001; Ori-Mckenney et al., 2012; Wang et al., 2014), and only γ-tubulin-associated Golgi elements were capable of MT nucleation during *in vitro* reconstitution assays (Chabin-Brion et al., 2001; Ori-Mckenney et al., 2012). In line with this, siRNA-driven depletion of γ-tubulin eliminates Golgi-derived MT nucleation in cells (Efimov et al., 2007). To understand regulation of MT nucleation at the Golgi, multiple investigators addressed a decisive question of γ-tubulin (or, rather, γ-tubulin ring complexes (γ-TuRC)) recruitment to the Golgi membrane. Several proteins with γ-TuRC-scaffolding capacity were identified in association with the Golgi membrane, including AKAP450 (also known as AKAP9, AKAP350, CG-NAP, Yotiao; Rivero et al., 2009), several isoforms of myomegalin (Roubin et al., 2013; Wang et al., 2014), CDK5Rap2 (Rios, 2014) or its homolog centrosomin in *Drosophila* neurons (Yalgin et al., 2015), and pericentrin in skeletal muscle (Oddoux et al., 2013). Also, PTTG/securin was identified as associated with this scaffolding complex (Moreno-Mateos et al., 2011). Depletion of each of these listed molecules has been demonstrated to attenuate Golgi-derived MT formation. Most significantly, AKAP450 has proven to be essential for Golgi-derived MT formation in multiple experimental systems and organisms (Rivero et al., 2009; Hurtado et al., 2011; Ori-Mckenney et al., 2012; Maia et al., 2013), likely because it can recruit γ-TuRC either directly, or indirectly via CDK5Rap2 or myomegalin interactions. However, AKAP450 function is not essential for MT nucleation at the Golgi in myotubes, likely because γ-TuRC is recruited to the Golgi by pericentrin in this cell type (Oddoux et al., 2013). So far, γ-TuRC-scaffolding to the Golgi has been attributed to proteins known as components of pericentrosomal matrix (see also Rios, 2014, for detailed review). The exception is a myomegalin splice-variant, MMG8 (Wang et al., 2014), which therefore might be important for specific regulation of MT nucleation at the Golgi. In any case, the pool of MT-nucleating factors in cells is likely restricted and has to be redistributed between the centrosome and the Golgi via regulated scaffolding. For example, release of centrosomal nucleation machinery by Cep192 depletion facilitates Golgi-derived nucleation (O'Rourke et al., 2014). This does not occur if the centrosomal nucleating complex is destroyed/denatured by laser ablation (Efimov et al., 2007).

This collected data prompted a widely accepted view that anchoring of γ-TuRCs at Golgi membranes is essential for Golgi-derived MT formation. The nucleation event might occur at the already Golgi-bound γ-TuRC; alternatively, MTs that are randomly nucleated at cytosolic γ-TuRCs in the vicinity of the Golgi membrane might be recruited to the Golgi thereafter. However, it cannot be overlooked that the concentration of γ-TuRCs at the Golgi is only slightly, if at all, higher than in the surrounding cytosol; it can only be detected on isolated Golgi membranes (Chabin-Brion et al., 2001; Ori-Mckenney et al., 2012), or after pre-extraction of cytosolic γ-tubulin (Wang et al., 2014), which is in sharp contrast to high γ-TuRC concentration in the pericentrosomal material. Since molecular anchoring does not concentrate γ-tubulin at the Golgi, it cannot be the reason for preferential nucleation of MTs at the Golgi as compared to other cytoplasmic sites, and the mechanism that allows the Golgi membrane to serve as a MTOC remains unclear. An attractive, though unexplored, possibility is that γ-TuRC structure and efficiency as a template requires specific stabilization/optimization of its structure by Golgi membrane-associated factors. Other mechanisms favoring MT nucleation at the Golgi might enhance MT formation at available templates. For example, Golgi environment could promote tubulin polymerization off the γ-TuRC templates and/or facilitate stabilization of MTs that already started to polymerize, preventing their immediate catastrophe. Such support of MT formation can be provided by MT plus tip tracking proteins (+TIPs) and stabilizing factors, if such factors are enriched at the MTOC. Indeed, similar to the centrosome-based MTOC, formation of Golgi-derived MT involves both types of molecular components. Below, we will briefly discuss the current knowledge on these two types of molecular components.

## Regulation of MT polymerization as a factor in Golgi-derived MT nucleation

While providing a template for MT nucleation is the initial condition for MT outgrowth, it has been recently proven to be insufficient for MT formation: additional factors are required to overcome the kinetic barrier and make MT nucleation kinetically favorable (Wieczorek et al., 2015). The process of “approval” of MT outgrowth from provided templates is referred to as “templated nucleation” and is tuned by MT +TIPs with variable activities.

One of the first MT-binding proteins implicated in MT nucleation at the Golgi is CLASP (Efimov et al., 2007), a known multi-functional MT stabilizer. CLASP functions include promotion of MT rescues, stabilization of MT seeds in fission yeast, capture of MTs at the cell cortex and kinetochores, and modification of polymerizing MT lattice (Galjart, 2005; Bratman and Chang, 2007; Kumar et al., 2009; Al-Bassam et al., 2010; Maia et al., 2012; Grimaldi et al., 2014). Both CLASP paralogs (CLASP1 and CLASP2 in mammals) strongly accumulate at the Trans-Golgi Network (TGN) membranes via scaffolding golgin GCC185, and are essential for efficient MT nucleation (Efimov et al., 2007). To date, the mechanistic role of CLASP in MT nucleation at the Golgi is not clear. We and others have proposed that CLASP stabilizes MTs as they start to form, based on the finding that newly-nucleated Golgi-derived MTs are coated with CLASP, which likely relocalized from the Golgi membrane. Now that CLASP is known to modify polymerizing MTs (Grimaldi et al., 2014), an alternative hypothesis arises that CLASP is essential for the initial polymerization steps of Golgi-derived MTs (templated nucleation), rather than simply stabilizing already assembled polymers. This function could be mediated through its TOG (tumor overexpressed gene) domains, which are MT-binding domains required for CLASP's MT-interaction (Slep, 2009; Maki et al., 2015). It is important in this regard that another TOG-domain containing protein, XMAP215, was recently shown to directly assist MT nucleation off a variety of templates (Wieczorek et al., 2015). Interestingly, another recent study indicates that XMAP215 synergizes in MT nucleation activity with TPX2 (Roostalu et al., 2015), a major stimulator of non-centrosomal MT formation in mitotic cells (Neumayer et al., 2014).

Furthermore, both Golgi-specific isoforms of myomegalin bind MT End Binding proteins (EB1 and/or EB3). These isoforms include MMG8, with both γ-TuRC and EB-binding motifs (Wang et al., 2014) and EB-MMG, with EB-binding motif only (Roubin et al., 2013). EBs are multi-functional + TIPs (Morrison, 2007; Slep, 2010), which cooperate with XMAP215 to promote MT polymerization (Zanic et al., 2013), actively regulate MT polymerization by modulating MT structure (Zhang et al., 2015), and are mutually regulated by CLASPs (Grimaldi et al., 2014). EB-binding myomegalins have recently emerged as critical regulators of MT nucleation at the Golgi (Roubin et al., 2013; Wang et al., 2014); it is possible that myomegalin interaction creates a local pool of EB molecules that can readily relocalize to MT-nucleating sites to support templated nucleation.

We suggest that regulation of Golgi-derived MTs at the level of templated nucleation at already available γ-TuRCs serves for fine modulation of this MT subpopulation because MT +TIPs are known as highly regulated by cell cycle and signaling cues. For example, both CLASP2 and EB1 are phosphorylated by cell cycle kinases (Maia et al., 2012; Banerjee et al., 2014). Also, the amount of CLASP2 at the Golgi membrane is tightly regulated by aPKC-driven phosphorylation (Matsui et al., 2015), indicating a potential link between polarity signaling and Golgi-derived MTs. Moreover, it was recently shown that during mitosis, one of the major Golgi components GM130 (among other molecules) is capable to promote TPX2-dependent MT nucleation, indicating a likely alternative mechanism of MT nucleation in the vicinity of Golgi membrane (Wei et al., 2015).

Besides MT + TIPs, an important factor that likely restricts MT nucleation efficiency of the Golgi is availability of functional alpha/beta tubulin dimers. Templated nucleation is kinetically favored at higher tubulin concentration than MT polymerization *per se* (Wieczorek et al., 2015). It has become clear recently that tubulin folding “quality control” performed by tubulin chaperones is important not only at the protein synthesis stage but also for tubulin recycling during dynamic MT reorganization (Nithianantham et al., 2015). One significant alpha-tubulin chaperone, TBCE, is concentrated at the Golgi membrane in an Arf1-regulated manner (Schaefer et al., 2007; Bellouze et al., 2014), and facilitates both nucleation rates and polymerization speed of Golgi-derived MTs (Bellouze et al., 2014). Because of low γ-TuRC abundance at the Golgi, nucleation of MTs off these γ-TuRCs templates may require high local concentration of functional tubulin dimers, which could be achieved by TBCE concentrating and reviving tubulin in the vicinity of nucleation sites. Regulation of TBCE via Arf1 activity (Bellouze et al., 2014) adds another level to potential signaling pathways that fine-tune the Golgi-derived MT population.

## Stabilization of Golgi-derived microtubules

MT function, in general, depends strongly on their lifespan, which can be extremely variable within a single cell and between different cell types. MTs associated with the Golgi are known to be more stable compared to the majority of cellular MTs, as has been detected both directly, in depolymerization resistance assays, and indirectly, via accumulated post-translational modifications of tubulin (see references below). A number of MT stabilizing factors have been identified as specifically active in the Golgi region. First of all, major Golgi-derived MT-promoting proteins AKAP450 and CLASP, which have been discussed above in conjunction with MT nucleation steps, are both capable of MT stabilization (Akhmanova et al., 2001; Larocca et al., 2006; Hurtado et al., 2011). Other factors, which specifically stabilize MTs in the Golgi region include: (1) recently identified microtubule cross-linking protein MTCL1, which interestingly can be recruited to the Golgi in both CLASP-dependent and AKAP450-dependent manner (Sato et al., 2013, 2014), (2) MT-stabilizing tumor suppressor RASSF1A (Arnette et al., 2014), (3) Golgi-anchored Cap-Gly-domain containing CAP350 (Hoppeler-Lebel et al., 2007), and (4) cytoskeletal linker dystonin, which localizes to the Golgi and nearby ER (Ryan et al., 2012) and stabilizes MTs in the Golgi area via interaction with MAP1b (Ryan et al., 2012).

In most studies, the origin of MTs, stabilized by specific proteins in the Golgi vicinity, has not been tested, and it would not be correct to imply that only MTs nucleated at the Golgi can be stabilized via these mechanisms. However, Golgi-derived MTs obviously fall into the category of MTs in the Golgi vicinity, and are stabilized by the described factors. Importantly, the pioneering study of the Pous group described rapid acetylation of newly-nucleated Golgi-derived MTs (Chabin-Brion et al., 2001), which suggests that these MTs can indeed be stabilized as soon as they form. Moreover, depletion of most MT stabilizers mentioned above, disturbs Golgi complex integrity (Hoppeler-Lebel et al., 2007; Oddoux et al., 2013; Arnette et al., 2014; Sato et al., 2014), a well-known function of Golgi-derived MTs (Miller et al., 2009; Vinogradova et al., 2012). Along the same lines, Golgi integrity also requires CAMSAP2 and 3 proteins (Tanaka et al., 2012), which specifically stabilize minus ends of MTs (Akhmanova and Hoogenraad, 2015) and have been recently implicated in stabilization and long lifespan of non-centrosomal MTs in neurons (Yau et al., 2014).

**Figure 1:**
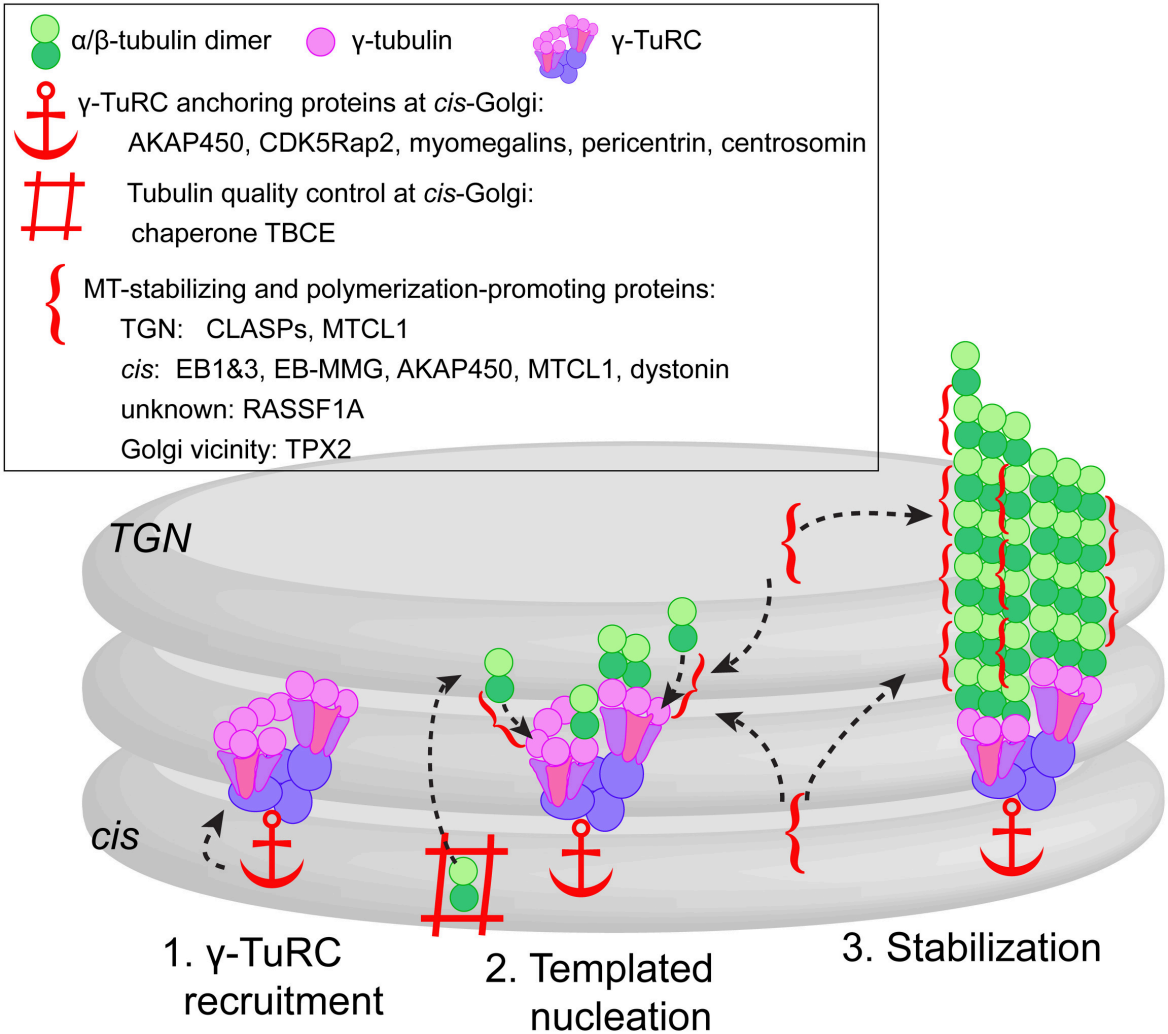
**Model of three-step MT formation at the Golgi**. (1) γ-TuRCs are recruited to the *cis*-Golgi membranes by scaffolding proteins, some of which might function as a complex (e.g., AKAP450, CDK5Rap2, and myomegalins) or as alternative cell type-specific mechanism (e.g., pericentrin). (2) Actual MT nucleation at γ-TuRC template is supported by tubulin pool provided by chaperone TBCE from the *cis*-face, and promoted by +TIPs (CLASPs, EBs concentrated by EB-MMG interaction) and MT stabilizing proteins (MTCL1, AKAP450, RASSF1A) localized at both *cis* and TGN faces of the Golgi. In mitosis, MT nucleation in the Golgi vicinity is facilitated by TPX2 (not depicted). (3) After successful nucleation event, the same set of +TIPs and MAPs promotes persistent growth and stabilizes emerging MTs.

Mechanisms of MT stabilization at the Golgi, which are obviously numerous and probably redundant, likely serve to extend Golgi-derived MT lifetime, allowing for their robust function. Thus, we consider MT stabilization as an important step in MT formation at the Golgi (Figure 1). That being said, while we have grouped known molecular factors in two groups based on existing evidence, it is possible that many MT stabilizers are capable of templated MT nucleation support, and vice versa. It is also possible that increased stability of these MTs is essential for their specific functions because MT-dependent molecular motors often sense post-translational modifications at stable MT lattice, which influences motor affinity and/or activity (Reed et al., 2006; Verhey and Hammond, 2009).

## Functional significance of MT anchoring at the Golgi

Evidence that factors localized both at the *cis*-Golgi (γ-TuRC scaffolding complex; Rivero et al., 2009) and at the TGN (CLASPs; Efimov et al., 2007) are required for Golgi-derived MT nucleation, present an interesting, as of yet, not fully answered question: Where exactly are Golgi-derived MTs nucleated? As mentioned above, localization of MT minus ends at the Golgi is likely important for specialization of MT function in trafficking, and association with a specific compartment would predict Golgi-derived MT application in trafficking to/from this Golgi domain. Live-cell imaging of new MT formation during nocodazole washout reveals existence of nucleation “hot spots” within a single Golgi stack (Efimov et al., 2007). It is possible that Golgi-derived MTs are nucleated at extensions of the *cis*-Golgi and TGN membranes, which bring two protein pools into close proximity. It is plausible to hypothesize that MTCL1, which interacts with both *cis*-localized AKAP450 and TGN-bound CLASP (Sato et al., 2014), plays a role in organization of nucleation “hot spots”; however, the nature of the exact Golgi-derived MT nucleation/trapping sites is yet to be determined.

From the functional point of view, the exact localization of MT-nucleating sites is important because, in many cases, it defines localization of MT minus ends, and thus directionality of MT-dependent transport. It is known that when γ-TuRC scaffolding factor ninein is absent from the centrosome, MT anchoring is compromised despite remaining γ-TuRC abundance. This scenario is utilized at the daughter centriole in G2 cells (Delgehyr et al., 2005), where MTs are nucleated but rapidly released. Thus, tight γ-TuRC anchoring at the *cis*-Golgi could serve as a mechanism for retaining MT minus ends at the *cis*-Golgi membrane and maintaining perfect positioning of MT minus ends in regard to ER-to-Golgi and Golgi-to-ER transport. Similarly, because MT nucleation requires proximity of the TGN-concentrated factors, MTs are likely readily positioned to the sites of TGN carrier formation (Luini et al., 2008), or late endosome-TGN recycling (Itin et al., 1999).

## Concluding remarks

As mentioned above, the vast majority of molecules involved in Golgi-derived MT formation are also accumulated in the pericentrosomal material and facilitate MT nucleation at the centrosome. Thus, an important factor of Golgi-derived MT nucleation is the balance between the two pools: depletion of centrosomal scaffolding factors results in the boost of Golgi MTOC activity (O'Rourke et al., 2014). Similarly, in differentiating myotubes, pericentrosomal protein complexes relocate to the Golgi simultaneously with the centrosome silencing (Zaal et al., 2011; Oddoux et al., 2013).

Another implication of the knowledge discussed above is that while anchoring of γ-TuRCs to the Golgi membrane is essential for the Golgi-derived MT array, MT nucleation at this location must be triggered at the level of subsequent steps of MT formation: tubulin polymerization off the template and/or MT lattice stabilization, as it was described for other non-centrosomal MT nucleation sites (Petry and Vale, 2015). We favor these views simply because MT +TIPs and MT stabilizers are strongly concentrated in the Golgi region, in contrast to γ-TuRCs.

Finally, it is plausible to suggest that specific anchoring sites of MT minus ends at the Golgi are important for their function as cellular roadways, and involvement of both *cis*- and TGN membranes in organization of the nucleation sites might serve to provide routes for both *cis*- and *trans*-Golgi associated trafficking.

### Conflict of interest statement

The authors declare that the research was conducted in the absence of any commercial or financial relationships that could be construed as a potential conflict of interest.
